# Mortality trend due to chronic kidney disease in Brazil: an ecological study

**DOI:** 10.1590/S2237-96222023000300010.EN

**Published:** 2023-11-27

**Authors:** Ellen de Cassia Dutra Pozzetti Gouvêa, Alex Mussoi Ribeiro, Erika Carvalho de Aquino, Sheila Rizzato Stopa

**Affiliations:** 1 Ministério da Saúde, Secretaria de Vigilância em Saúde e Ambiente, Brasília, DF, Brazil; 2 Universidade Federal de Santa Catarina, Programa de Pós-Graduação em Controle de Gestão, Florianópolis, SC, Brazil; 3 Ministério da Saúde, Secretaria de Vigilância em Saúde e Ambiente, Brasília, DF, Brazil; 4 Autonomous researcher, Epidemiology, Brasília, DF, Brazil

**Keywords:** Chronic Kidney Disease, Mortality, Time Series Studies, Epidemiology, Enfermedad Renal Crónica, Mortalidad, Estudios de Series Temporales, Epidemiología, Doença Renal Crônica, Mortalidade, Estudos de Séries Temporais, Epidemiologia

## Abstract

**Objective:**

To analyze chronic kidney disease mortality in Brazil according to sex, age group and region of residence, from 2009 to 2020.

**Methods:**

This was a time series study having deaths as its unit of analysis, based on Mortality Information System data. The mortality rate was standardized using the direct method and the temporal trend was analyzed using the Prais-Winsten method.

**Results:**

There was a rising trend in chronic kidney disease mortality, ranging from 2.82, in 2009, to 3.24 in 2020 (average annual increase 1.29%; 95%CI 0.73;1.85), with a greater increase in males (1.14% per year; 95%CI 0.52;1.76), those aged 75 years and over (2.23% per year; 95%CI 1.87; 2.60) and in the Northern Region (3.86% per year; 95%CI 1.86;5.90) and Northeast Region (3.36% per year; 95%CI 2.24;4.50).

**Conclusion:**

Chronic kidney disease mortality showed a rising trend in the period, with sociodemographic disparities.

## Study contributions

**Main results:**

A rising mortality trend was found for both sexes, with a greater increase in males, those aged over 75 years and in the North and Northeast regions of Brazil; mortality was highest in the Midwest region throughout the entire period.

**Implications for services:**

The results point to the need to implement public policies with guidelines for addressing chronic kidney disease, focused on strengthening Primary Health Care (PHC).

**Perspectives:**

Expanding access to health services, health education and integration between PHC, health surveillance and specialized care are strategies that would possibly prove to be efficient in managing this chronic health condition.

## RESUMEN

**Objetivo:**

Analizar la mortalidad por enfermedad renal crónica en Brasil según sexo, grupo de edad y región de residencia, de 2009 a 2020.

**Métodos:**

Estudio de serie temporal con óbitos como unidad de análisis, utilizando el Sistema de Información sobre Mortalidad. La tasa de mortalidad se estandarizó por el método directo y la tendencia temporal se analizó por el método de Prais-Winsten.

**Resultados:**

Tendencia creciente de la mortalidad por enfermedad renal crónica, variando de 2,82, en 2009, a 3,24 en 2020 (incremento promedio anual 1,29%; IC_95%_ 0,73;1,85), con mayor aumento en el sexo masculino (1,14% por año; IC_95%_ 0,52;1,76), con 75 años y más (2,23% por año; IC_95%_ 1,87;2,60) y en las Regiones Norte (3,86% por año; IC_95%_ 1,86;5,90) y Nordeste (3,36% por año; IC_95%_ 2,24;4,50).

**Conclusión:**

La mortalidad por enfermedad renal crónica mostró una tendencia creciente en el período, con disparidades sociodemográficas.

## RESUMO

**Objetivo:**

Analisar a mortalidade por doença renal crônica no Brasil segundo sexo, faixa etária e região de residência, no período de 2009 a 2020.

**Métodos:**

Estudo de série temporal, tendo os óbitos como unidade de análise, utilizando-se o Sistema de Informação sobre Mortalidade. A taxa de mortalidade foi padronizada pelo método direto, e a tendência temporal analisada pelo método de Prais-Winsten.

**Resultados:**

Tendência crescente da mortalidade por doença renal crônica, variando de 2,82, em 2009, para 3,24 em 2020 (incremento médio anual de 1,29%; IC_95%_ 0,73;1,85), com maior incremento no sexo masculino (1,14% ao ano; IC_95%_ 0,52;1,76), na faixa etária com 75 anos e mais (2,23% ao ano; IC_95%_ 1,87;2,60), nas regiões Norte (3,86% ao ano; IC_95%_ 1,86;5,90) e Nordeste (3,36% ao ano; IC_95%_ 2,24;4,50).

**Conclusão:**

A mortalidade por doença renal crônica apresentou tendência crescente no período, com disparidades sociodemográficas.

## INTRODUCTION

Chronic kidney disease (CKD) is an important direct cause of death and a risk factor for several health problems,^1^ ^),(^ ^2^ ^)^ especially cardiovascular problems.^3^ CKD has economic impacts and directly affects the quality of life of those who have this condition.^4^ ^),(^ ^5^ ^)^

Worldwide, it is estimated that approximately 850 million people have kidney disease.^1^ In 2019, kidney disease was responsible for 3.16 million deaths globally.^6^ ^),(^ ^7^ From 1990 to 2017, global mortality from chronic non-communicable diseases (NCDs) decreased; however, a similar decline in CKD was not observed in the same period.^8^ Estimates also indicate that 7.6% of all deaths from cardiovascular disease (CVD) that occurred in 2017 are - probably - related to changes in kidney function;^3^ when taken together, deaths from CKD or CVD attributed to CKD represented 4.6% of deaths from all causes.^3^ CKD is defined as kidney damage capable of affecting both kidney structure and function and/or glomerular filtration rate, regardless of the cause.^4^ It is characterized as evolving in a silent, progressive and irreversible manner.^5^ ^),(^ ^9^

Progression of CKD to more advanced stages leads to the need for renal replacement therapy (hemodialysis, peritoneal dialysis and kidney transplant), in order to preserve the lives of those with this condition, although at a high cost to health systems.^1^ Furthermore, CKD progression contributes to a decrease in quality of life, in addition to favoring the occurrence of cardiovascular complications.^10^ ^)^

In Brazil, between 2009 and 2019 CKD mortality increased by around 40%, rising from eleventh to ninth position among causes of death, especially among the elderly.^11^

In this context, identifying the profile of the Brazilian population at risk proves to be an important strategy in the implementation of public policies aimed at addressing CKD. 

This study aimed to analyze the (CKD) mortality trend in Brazil, according to sex, age group and macro-region of residence, from 2009 to 2020.

## METHODS

*Study design*

This was an ecological time series study, with analysis of CKD mortality in Brazil, according to sex, age group and region of residence in the country, from 2009 to 2020, using recorded deaths as the unit of analysis. 

*Context*

Brazil consists of 5,570 municipalities, subdivided into 27 Federative Units distributed over five national macro-regions: North, Northeast, Southeast, South and Midwest. In 2022, the country had 203,062,512 inhabitants, with the Southeast region being the most populous, with 84.8 million inhabitants, or 41.8% of the Brazilian population, followed by the Northeast (26.9%), South (14.7%), North (8.5%) and Midwest (8.0%) regions.^12^ ^)^ Considering the regional diversities of the Brazilian population, evaluating the mortality rate stratified by sex, age and region of residence contributes to knowledge about the epidemiological profile of the population, by showing the evolution and enabling comparison of the level of health over time and consequently, assisting in planning actions to address the health condition analyzed.^13^

As such, in June 2021, CKD mortality data was collected from the Mortality Information System (Sistema de Informações sobre Mortalidade - SIM). These are data relating to deaths that occurred in the period from 2009 to 2019; in May 2022, the data collected was updated by adding records of occurrences in 2020. Analyses were then performed. 

*Participants*

Deaths which had CKD as the underlying cause recorded on the Death Certificates, identified by the corresponding code N18 of the Tenth Revision of the International Statistical Classification of Diseases and Related Health Problems (ICD-10) were included in the analysis. 

*Variables*

The annual mortality rate was calculated, per 100,000 inhabitants, standardized by age. This indicator was calculated according to sex (male; female), age group (in years: less than 1; 1-4; 5-14; 15-24; 25-34; 35-44; 45-54; 55-64; 65-74; 75 and over), year of death (between 2009 and 2020) and macro-region of residence (North; Northeast; South; Southeast; Midwest). 

*Data collection*

We used (i) data from SIM and (ii) annual estimates of resident population, according to the the Brazilian Institute of Geography and Statistics (Instituto Brasileiro de Geografia e Estatística - IBGE), both available on the website of the National Health System Information Technology Department (Departamento de Informática do Sistema Único de Saúde - DATASUS).^14^

*Data analysis*

Standardization was carried out using the direct method, taking the Brazilian population in 2010 as the standard. The direct method guarantees the comparison of indicators throughout the period and between the geographic units studied. To calculate specific standardized mortality by sex and age group, data entered as unknown were excluded from the analyses.

Temporal trends were estimated using the Prais-Winsten method for generalized linear regression. This is a method suitable for analyzing data that can be influenced by serial autocorrelation, which allows value of the regression slope coefficient to be estimated. Linear autocorrelation breaks with one of the main premises of simple linear regression analysis: the independence of residuals.^15^ A significance level of p-value = 0.05 was adopted as a critical value for trend analysis. Average annual increase was calculated using the following formula:^15^ ^)^

Average annual increase = a+10^b

where “a” corresponds to the mortality value in year zero of the series (intersection between the X and Y axes) and “b” corresponds to the slope coefficient of the line obtained in the regression analysis. The 95% confidence interval of the average annual percentage increase in the period was calculated using the following formula:^15^ ^)^

95%CI = -1+10^[(b ± t*EP)]

where “t” is the value at which Student’s t distribution has 11 degrees of freedom, at a two-tailed 95% confidence level, and “EP” is the standard error of the estimate of “b” provided by the regression analysis. The analyses were performed using Stata 14.0 software (StataCorp. 2015. Stata Statistical Software: Release 14. College Station, TX: StataCorp LP). Annual increase was calculated using Microsoft Excel 2007. 

*Ethical aspects*

This study was not submitted to a Research Ethics Committee because the databases used are publicly accessible and anonymized.

## RESULTS

Between 2009 and 2020, 81,034 CKD deaths were recorded in Brazil. The majority of deaths occurred among males (57.4%), with emphasis on the 75 years and over age group (43.1%). Regarding age, an increase in the proportion of deaths was observed as age increased. The Southeast region recorded the highest number of occurrences: 47.5% (Table 1). 

When calculating sex-specific standardized mortality, 9 deaths were excluded from the analysis, while 35 were excluded in relation to age group, as information in these fields had been recorded as “unknown”. 

**Figure 1:**
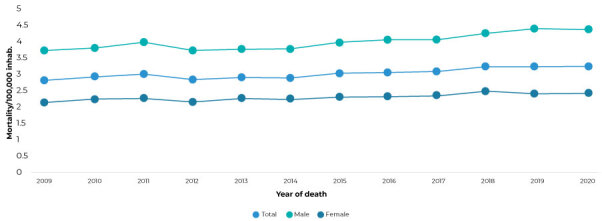
Chronic kidney disease mortality rate by sex,^a^ Brazil, 2009-2020

CKD mortality in Brazil, standardized according to sex, in the period from 2009 to 2020 (Figure 1), was higher among males; however, there was an increasing trend for both sexes.

**Figure 2:**
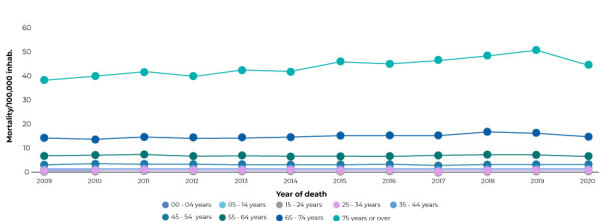
Chronic kidney disease mortality rate according to victims’ age group,^a^ Brazil, 2009-2020

The highest mortality rate were found in the older age groups. The age range of 75 and over had the highest CKD mortality rate, while those aged 65 to 74 came in second place, in relation to the other age groups, throughout the entire period (Figure 2).

The CKD mortality rate according to geographic region of the country was higher among residents of the Midwest throughout the entire period analyzed. As of 2015, the Northern region came in second place. The Northeast region had the lowest mortality rates from 2009 to 2013, returning to this position after 2018. In the period from 2014 to 2017, the Southern region had the lowest mortality rates among all regions (Figure 3).

The analysis showed an increasing trend in the CKD mortality rate for Brazil as a whole, from 2009 to 2020, varying from 2.82 in 2009 to 3.24 in 2020 (average annual increase of 1.29%; 95%CI 0.73 ;1.85), for both sexes, for age groups over 75 years old and for the North and Northeast regions. Only the 35-44 age group showed a falling trend; while among the other age groups the trend was stationary (Table 2).

**Table 1:** Sociodemographic characteristics of deaths due to chronic kidney disease (N = 81,034), Brazil, 2009-2020

Year of death	2009		2010		2011		2012		2013		2014		2015		2016		2017		2018		2019		2020		Total	
	n	%	N	%	n	%	n	%	n	%	N	%	N	%	N	%	N	%	N	%	N	%	N	%	N	%
Sex																										
Male	3,045	58.0	3,222	57.3	3,458	57.9	3,352	57.6	3,483	56.4	3,592	56.7	3,931	57.1	4,123	57.6	4,277	57.3	4,61	56.7	4,916	58.2	4,485	57.8	46,494	57.4
Female	2,2	42.0	2,4	42.7	2,518	42.1	2,472	42.4	2,695	43.6	2,743	43.3	2,954	42.9	3,04	42.4	3,191	42.7	3,514	43.2	3,526	41.8	3,278	42.2	34,531	42.6
Unknown	1	-	-	-	-	-	-	-	-	-	3	-	-	-	1	-	2	-	2	-	-	-	-	-	9	-
Age group (in years)																										
< 1	4	0.1	10	0.2	4	0.1	3	0.1	2	-	3	-	5	0.1	2	-	4	0.1	10	0.1	8	0.1	2	-	57	0.1
01-04	9	0.2	10	0.2	5	0.1	12	0.2	9	0.1	8	0.1	9	0.1	11	0.2	6	0.1	10	0.1	8	0.1	5	0.1	102	0.1
05-14	26	0.5	17	0.3	24	0.4	24	0.4	13	0.2	9	0.1	22	0.3	25	0.3	20	0.3	10	0.1	13	0.2	17	0.2	220	0.3
15-24	74	1.4	76	1.4	77	1.3	71	1.2	57	0.9	80	1.3	62	0.9	70	1.0	61	0.8	81	1.0	70	0.8	81	1.0	860	1.1
25-34	156	3.0	184	3.3	144	2.4	133	2.3	149	2.4	140	2.2	147	2.1	151	2.1	165	2.2	154	1.9	133	1.6	157	2.0	1,813	2.2
35-44	299	5.7	284	5.1	317	5.3	297	5.1	300	4.9	320	5.0	311	4.5	340	4.7	336	4.5	312	3.8	328	3.9	343	4.4	3,787	4.7
45-54	602	11.5	674	12.0	659	11.0	640	11.0	624	10.1	580	9.2	631	9.2	714	10.0	633	8.5	675	8.3	679	8.0	666	8.6	7,777	9.6
55-64	909	17.3	985	17.5	1,067	17.9	982	16.9	1,054	17.1	1,037	16.4	1,093	15.9	1,112	15.5	1,224	16.4	1,338	16.5	1,359	16.1	1,27	16.4	13,43	16.6
65-74	1,145	21.8	1,166	20.7	1,275	21.3	1,26	21.6	1,32	21.4	1,428	22.5	1,524	22.1	1,604	22.4	1,674	22.4	1,921	23.6	1,933	22.9	1,756	22.6	18,006	22.2
≥ 75	2,015	38.4	2,212	39.3	2,402	40.2	2,4	41.2	2,645	42.8	2,732	43.1	3,08	44.7	3,128	43.7	3,345	44.8	3,611	44.4	3,911	46.3	3,466	44.6	34,947	43.1
Age unknown	7	0.1	4	0.1	2	-	2	-	5	0.1	1	-	1	-	7	0.1	2	-	4	-	-	-	-	-	35	0.0
Region of residence																										
North	310	5.9	312	5.5	363	6.1	345	5.9	331	5.4	383	6.0	409	5.9	470	6.6	552	7.4	584	7.2	659	7.8	591	7.6	5,309	6.6
Northeast	1,051	20.0	1,087	19.3	1,336	22.4	1,276	21.9	1,409	22.8	1,493	23.6	1,633	23.7	1,794	25.0	1,831	24.5	1,91	23.5	2,042	24.2	1,825	23.5	18,687	23.1
Southeast	2,608	49.7	2,891	51.4	2,903	48.6	2,904	49.9	3,045	49.3	3,048	48.1	3,304	48.0	3,389	47.3	3,518	47.1	3,765	46.3	3,763	44.6	3,382	43.6	38,52	47.5
South	868	16.5	908	16.2	959	16.0	867	14.9	994	16.1	920	14.5	1,032	15.0	1,087	15.2	1,099	14.7	1,351	16.6	1,328	15.7	1,389	17.9	12,802	15.8
Midwest	409	7.8	424	7.5	415	6.9	432	7.4	399	6.5	494	7.8	507	7.4	424	5.9	470	6.3	516	6.3	650	7.7	576	7.4	5,716	7.1
TOTAL	5,246	100.0	5,622	100.0	5,976	100.0	5,824	100.0	6,178	100.0	6,338	100.0	6,885	100.0	7,164	100.0	7,47	100.0	8,126	100.0	8,442	100.0	7,763	100.0	81,034	100.0

Source: General Coordination of Epidemiological Information and Analysis, Health and Environment Surveillance Secretariat, Ministry of Health (CGIAE/SVSA/MS); Mortality Information System (Sistema de Informações sobre Mortalidade - SIM).

a) Mortality/100,000 inhabitants, standardized by age.

Note: standard population - 2010 Demographic Census/Brazilian Institute of Geography and Statistics (Instituto Brasileiro de Geografia e Estatística).

a) Mortality/100,000 inhabitants, standardized by age.

Note: standard population - 2010 Demographic Census/Brazilian Institute of Geography and Statistics (Instituto Brasileiro de Geografia e Estatística).

**Figure:**
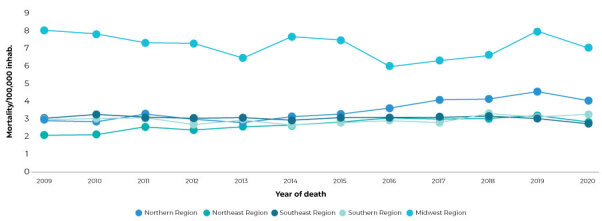
3 - Chronic kidney disease mortality rate by victims’ region of residence,^a^ Brazil, 2009-2020

a) Mortality/100,000 inhabitants, standardized by age.

Note: standard population - 2010 Demographic Census/Brazilian Institute of Geography and Statistics (Instituto Brasileiro de Geografia e Estatística).

**Table 2:** Values and analysis of the trenda of the chronic kidney disease mortality rate, Brazil, 2009-2020

Disaggregation	Mortality coefficient			Beta^b^	p-value	Average annual rate of increase (%)	95%CI^c^	Interpretation
	2009	2015	2020					
Sex								
Male	3.72	3.99	3.98	0.005	0.002	1.14	0.52;1.76	Rising
Female	2.13	2.30	2.23	0.004	0.008	0.82	0.28;1.37	Rising
Age group (in years)								
≤ 4	0.08	0.09	0.05	0.002	0.768	0.54	-3.34;4.58	Stationary
5-14	0.08	0.07	0.06	-0.012	0.385	-2.82	-9.32;4.15	Stationary
15-24	0.21	0.18	0.24	0.000	0.888	0.10	-1.46; 1.69	Stationary
25-34	0.47	0.42	0.46	-0.003	0.527	-0.58	-2.50; 1.38	Stationary
35-44	1.12	1.04	1.04	-0.003	0.018	-0.79	-1.40; -0.17	Falling
45-54	2.78	2.58	2.54	-0.004	0.094	-0.95	-2.07; 0.18	Stationary
55-64	6.49	6.21	6.17	-0.001	0.664	-0.23	-1.33;0.89	Stationary
65- 74	13.61	14.66	14.33	0.006	0.001	1.42	0.78;2.06	Rising
≥ 75	37.95	45.85	44.74	0.010	< 0.001	2.23	1.87; 2.60	Rising
Region of residence								
North	2.92	3.30	4.07	0.016	0.002	3.86	1.86;5.90	Rising
Northeast	2.12	2.83	2.83	0.014	< 0.001	3.36	2.24;4.50	Rising
Southeast	3.04	3.10	2.77	-0.002	0.187	-0.41	-1.05;0.23	Stationary
South	3.02	2.85	3.28	0.003	0.309	0.76	-0.80;2.35	Stationary
Midwest	7.99	7.47	7.05	-0.004	0.297	-0.92	-2.75;0.93	Stationary
TOTAL	2.82	3.03	3.24	0.006	< 0.001	1.29	0.73;1.85	Rising

a) Prais-Winsten Regression; b) Straight line slope coefficient; c) 95%CI = 95% confidence interval.

## DISCUSSION

In Brazil, from 2009 to 2020, there was an increasing trend in the CKD mortality rate for both sexes, more evident in the 75 years and over age group and in the North and Northeast macro-regions of the country. The study showed that in the Midwest region, the number of deaths was higher throughout the period, although the mortality rate showed a stationary trend. A falling mortality rate trend only stands in the case of 35-44 age group, while for the other age ranges the data demonstrated stability. 

Because CKD is a consequence of several conditions that lead to death, such as hypertension and diabetes *mellitus*, its mortality is underrated as an underlying cause of death and recorded with low frequency in official statistics.^16^ In this context, considering the rising mortality trend in the period studied, it is possible to associate this with improvement in the quality of filling out the underlying cause of death on Death Certificates.^17^ It is important to keep in mind that the magnitude of CKD mortality may be more expressive than presented in this analysis, in view of underreporting.

In this analysis, CKD mortality was higher among males, this being a result consistent with the literature.^1^ ^),(^ ^18^ However, in Australia and New Zealand, mortality is higher in females, compared to males.^19^ The difference in CKD mortality between the sexes in Brazil can be attributed to the fact that women seek health services more, whether for preventive consultations or for tests.^20^ Risk behaviors, such as inadequate nutrition, alcohol use and smoking, can also explain higher male mortality.^20^

More than two-thirds of CKD deaths occurred in age groups over 65 years old. Although CKD can affect individuals of any age, elderly people and those with associated comorbidities are at greater risk.^2^ ^),(^ ^19^ This finding may also reflect health service accessibility, quality of care, or even changes in life expectations and living conditions of the Brazilian population. 

The Midwest region had the highest mortality throughout the period studied, followed by the Northern region from 2015 onwards. In a study carried out over the period from 2008 to 2016, the Midwest and Northern regions had a lower proportion of hospitalizations due to CKD, suggesting inequalities in the provision of health care services between regions,^21^ also reflected in mortality. 

The North and Northeast showed an increasing trend in the CKD mortality rate in the period analyzed, while the trend was stable in the South and Southeast. Historically, the North and Northeast regions have the lowest socioeconomic indicators in Brazil,^22^ and this can affect mortality. In an analysis carried out by Baptista and Queiroz (2019),^23^ these same regions showed an increase in CVD and other NCD mortality in the period from 2001 to 2015. These data may be related to the aging of the population, health care accessibility, care network structure, among other socioeconomic issues in the Brazilian regions.^17^ The finding corroborates the analysis carried out on the epidemiological profile of health service users undergoing renal replacement therapy in Brazil, whereby the Southeast region had the highest rate from 2010 to 2017.^24^

According to a survey on CKD carried out in 167 countries, both mortality and morbidity have increased worldwide.^19^ Between Brazil, Russia, India, China and South Africa (BRICS), age-standardized CKD mortality in 2017, according to the Global Burden of Disease Study (GBD), was highest in India (22.3 per 100,000 inhab.) and South Africa (22.3 per 100,000 inhab.), followed by Brazil (16.1 per 100,000 inhab.). Also according to the GBD study, among the South American countries that took part in it, Ecuador had the highest mortality rate (40.2 per 100,000 inhab.) while Uruguay had the lowest rate (12.9 per 100,000 inhab.) and Brazil came in tenth position.^3^ ^)^ Despite rising mortality due to CKD, this problem is often not included in the main chronic disease control strategies, this being an obstacle to addressing and controlling CKD.^1^

Studies suggest that limited access to renal replacement therapy, both for starting care and for continuing with it, combined with prevalence of diabetes *mellitus* and hypertension, has contributed to increased CKD mortality.^3^ ^),(^ ^19^ Additionally, there is a gap between the total number of people with CKD and those who have access to renal replacement therapy services.^1^ Although renal replacement therapy prevents imminent death in people with advanced CKD, those treated with dialysis are at greater risk of death than the general population, mainly due to cardiovascular conditions.^19^ Providing renal replacement therapy alone does not guarantee a reduction in mortality. It is necessary to undertake government actions with guidelines on addressing CKD determinants and conditioning factors, focusing mainly on strengthening Primary Health Care.

The advent of the COVID-19 pandemic in 2020 may have influenced CKD mortality. Even though all populations are vulnerable to SARS-CoV-2 infection, individuals with pre-existing chronic conditions are more likely to have severe COVID-19 outcomes^25^ or it may or even worsen pre-existing conditions.^26^

Mitigating progression of CKD to a more advanced stage and reducing mortality depends on timely interventions, screening for risk factors and quality of care received at all stages of the disease (asymptomatic, pre-dialysis, dialysis). The National Policy on Care for People with Kidney Disease (Política Nacional de Atenção ao Portador de Doença Renal), established by the Ministry of Health on June 15, 2004, through the official publication of Ordinance No. 1,16827 and Ordinance No. 389, dated March 13, 2014, which defines the criteria for organization of the line of care for people with CKD,^28^ and more recently, the Line of Care for CKD,^29^ are legal frameworks the main objective of which is to guarantee comprehensive care, through health promotion, protection, recovery and rehabilitation, which permeates all levels of health care. The role of Primary Health Care in longitudinal monitoring and coordination of care together with specialized care is highlighted, whether in the identification, guidance and management of care for people with CKD and determinant and conditioning factors, or in setting the direction to be taken by the Health Care Network.^29^

The limitations of this study must be considered when interpreting the results. Secondary mortality and demographic data were used, and national mortality databases may have patchy coverage; underreporting may occur, in different proportions, between locations in the country, resulting in underestimated mortality.^17^ Another important limitation of this study is that based on the SIM, it is not possible to identify the stage of the disease (renal function) or the phase (dialysis or not). Inaccuracies when filling out Death Certificates may compromise the calculation of mortality. However, the SIM has good coverage in Brazil^17^ ^),(^ ^30^ and therefore, public and facilitated access to this data can be considered a positive point. 

An increase in CKD mortality can be seen in Brazil over the years, as well as demographic disparities and a greater risk of this event in males and the elderly. The results found by this study demonstrate the need to look at CKD from a different angle, especially in Primary Health Care, with a view to improving early identification strategies. Furthermore, raising awareness among the population and initial and continuing education of health professionals about CKD and risk factors can contribute to improving the scenario presented.

Care offered to people with CKD should not be restricted to providing renal replacement therapy. In addition to timely provision of renal replacement therapy, identification of individuals at risk of developing kidney disease should be mainly focused on early detection and comprehensive and longitudinal care to mitigate its progression. Integration between primary care services, health surveillance and specialized care is a strategy that may prove to be efficient in the management of CKD. 

The CKD is a factor that contributes to morbidity and mortality due to chronic non-communicable conditions, and addressing it effectively can contribute to Brazil achieving target 3.4 of the Sustainable Development Goals (SDGs), in the sense of meeting the provisions of the United Nations 2030 Agenda: By 2030, reduce by one third premature mortality from non-communicable diseases.
